# Sources of seismic noise in an open-pit mining environment

**DOI:** 10.1038/s41598-024-75733-2

**Published:** 2024-10-15

**Authors:** J. Diaz, M. Torne, M. Schimmel, S. Rodríguez, D. Martí, M. Ruiz, H. Seivane, P. Sánchez-Pastor, D. Davoise

**Affiliations:** 1 Geosciences Barcelona (GEO3BCN), CSIC, c. Solé Sabarís sn, Barcelona, 08028 Spain; 2 Atalaya Mining, La Dehesa s/n. 21660 Minas de Riotinto, Huelva, Spain; 3 Instituto Geológico y Minero de España (IGME-CSIC), Madrid, Spain

**Keywords:** Seismology, Civil engineering

## Abstract

We present the characterization of the sources of vibration in the open-pit Riotinto mine (southern Spain), based on the data recorded by a dense seismic network of 30 stations located along a 1-km long segment of a tailings dam. We describe the most common transient signals detected, including local and distant earthquakes, blasting and vehicles. The time variations in the amplitude of the 10–40 Hz frequency band are then used to define three phases of activity during the recording period. The phase with the highest seismic amplitudes is observed during a time interval of five weeks, coinciding with the civil works carried out for the regrowth of the dam, necessary to correctly capture the continuously increasing amount of tailings. In the last three months of operation of our network, the seismic noise is dominated by the deposition of tailings into the pond, enabling the use of seismic data to monitor into detail the evolution of the deposition process.

**Supplementary Information:**

The online version contains supplementary material available at 10.1038/s41598-024-75733-2.

## Introduction

In the last decade, seismic imaging using ambient noise data, the vibrations recorded in the absence of waves generated by earthquakes, has become one of the most powerful tools to image the Earth’s interior and, in particular, the uppermost levels of the crust. When compared to other seismic methodologies, this approach offers the advantages of reduced costs, low social and environmental impact and independence of the occurrence and spatial distribution of seismicity. Different methods can be applied, including the use of the dispersion curves of the surface waves extracted from the correlation of seismic noise to obtain tomographic images at different depths^1^ or the analysis of amplitude variations at certain frequency bands^2^. Ambient seismic noise has also been widely used for monitoring, as waveform variations in the coda of seismic ambient noise correlations obtained at different time lapses can be related to changes in the physical properties of the media^3–5^ However, the presence of varying but persistent noise sources in the frequency band of interest may challenge the application of ambient noise seismic interferometry (ANSI) techniques^6,7^, as it may difficult to discern between changes due to structural modifications and changes due to source variability. This issue becomes more difficult to cope in areas with high activity such as cities, mines, geothermal fields, active volcanoes, etc. where there are numerous transient noise sources in a broad frequency band. The objective of this contribution is to characterize the sources of seismic noise in an active open-pit mining environment, identifying the contribution of transient sources, as heavy service vehicles, engineering activities, machinery etc. and time sustained noise sources. The interest of this study is twofold; firstly, to get a catalogue of noise sources in a mining environment and secondly to prevent eventual misinterpretations in a forthcoming analysis of time-varying physical properties using ANSI methods. In addition, the results can be used to train machine learning procedures designed to improve the monitoring of seismic activity due to mining activity.

The present study is being performed within the framework of the STONE project (Smart Terrain Control Using Cutting-Edge Technologies at the Rio Tinto mine, Spain). The project is a public-private collaboration project between two institutes of the Spanish National Research Council (Consejo Superior de Investigaciones Científicas, CSIC), the Institute of Geosciences (IGEO-UCM-CSIC) and Geosciences Barcelona (GEO3BCN-CSIC) and the Atalaya Riotinto Minera S.L. It aims to create a multidisciplinary monitoring and interpretation platform that, in real time, centralizes the observations obtained with classical and state-of-the-art monitoring techniques to reduce risk and improve safety of the tailings dam of the Riotinto mine, located in the province of Huelva (SW Spain). One of the specific objectives is to explore the applicability of ANSI, along with Interferometric Synthetic Aperture Radar (InSAR), as operational monitoring technologies in a mining environment, transferring the knowledge and applicability of both techniques from academia to industry. Although some contributions have already shown the potential of InSAR^8^ and ambient seismic noise^9^ methods in mining environments, we expect that the pioneering joint interpretation of both approaches in operational mines may mark a pivotal moment in contemporary monitoring practices, facilitating the shift toward “Smart mining” and enhancing the safety of both equipment and personnel within mining operations.

The Riotinto mine is an open-pit mine within the highly productive Iberian Pyrite Belt, a region recognized for having one of the largest concentrations of massive sulfides in the world. The mine has a long exploitation history dating back to the Roman period and is currently operating as a fully functional open-pit copper mine owned by Atalaya Mining (https://atalayamining.com/, last accessed 25/9/2024). Comprehensive studies have been conducted to establish a robust, long-term mining project to exploit its copper ore reserves, which amount to 176 million tons and contain 651,000 tons of copper metal. This equates to an average copper grade of 0.37%. The mining operation processes 15.5 million tons of ore annually, with a cut-off grade of 0.14%. The mine complex covers an approximate area of 35 km^2^ and is divided into three main sectors: the southern sector, which includes two benches with the corresponding waste dumps; the central sector, housing the crushing and processing plant support facilities such as offices, maintenance workshops, etc.; and the northern sector, primarily occupied by the tailings ponds and the clean water area. Atalaya mining tailings ponds consist of three deposits -the Gossan, Cobre, and Aguzadera- located in the Odiel river basin. Two water reservoirs – the Campofrío and Aguas Limpias – are located upstream of the tailings deposit and are drained by a perimeter channel. Atalaya’s tailings dams are classed as Category A dams under the Basic Directive on Civil Protection 1994, meaning that failure or malfunction of the dams may seriously affect urban centers or essential services, or produce very significant material or environmental damage. Therefore, safety has always been of utmost importance when designing, constructing, and expanding tailings ponds at the Riotinto mine.

As different modes of failures can affect tailings dams^10^, it is important to implement safety monitoring programs based on different methodologies^11^. The tailings ponds in the Riotinto mine are currently monitored by an extensive network of geodetic and geotechnical sensors. For geodetic control, 4 GNSS GMX units have been installed, and their positions are autonomously and continuously calculated in real time with millimetric precision in relation to an established and external geodetic station at the mine. Additionally, for geotechnical monitoring, the installed instrumentation includes 47 open piezometers, 30 closed piezometers, 21 inclinometers, 124 prisms, 25 benchmarks, and 1 remote ground-based radar sensor (GBSAR IBIS FME EVO), providing discrete measurements with high accuracy.

Previous works have shown that ambient noise can be used to investigate subsurface changes nearby tailings dams^9^. A final objective of the STONE project is the combination of satellite radar and ambient seismic noise interferometry, which can significantly complement classical observation techniques and help reduce the inherent risk in mining environments, since they allow extensive monitoring, covering areas where other sensors do not reach (e.g., intermediate and low priority areas, areas of difficult access or peripheral areas). In addition, both are non-invasive tools, allow great repeatability, are low cost, can be automated and require low maintenance, all of which are ideal characteristics for a monitoring system.

## Data and methods

**Fig. 1:**
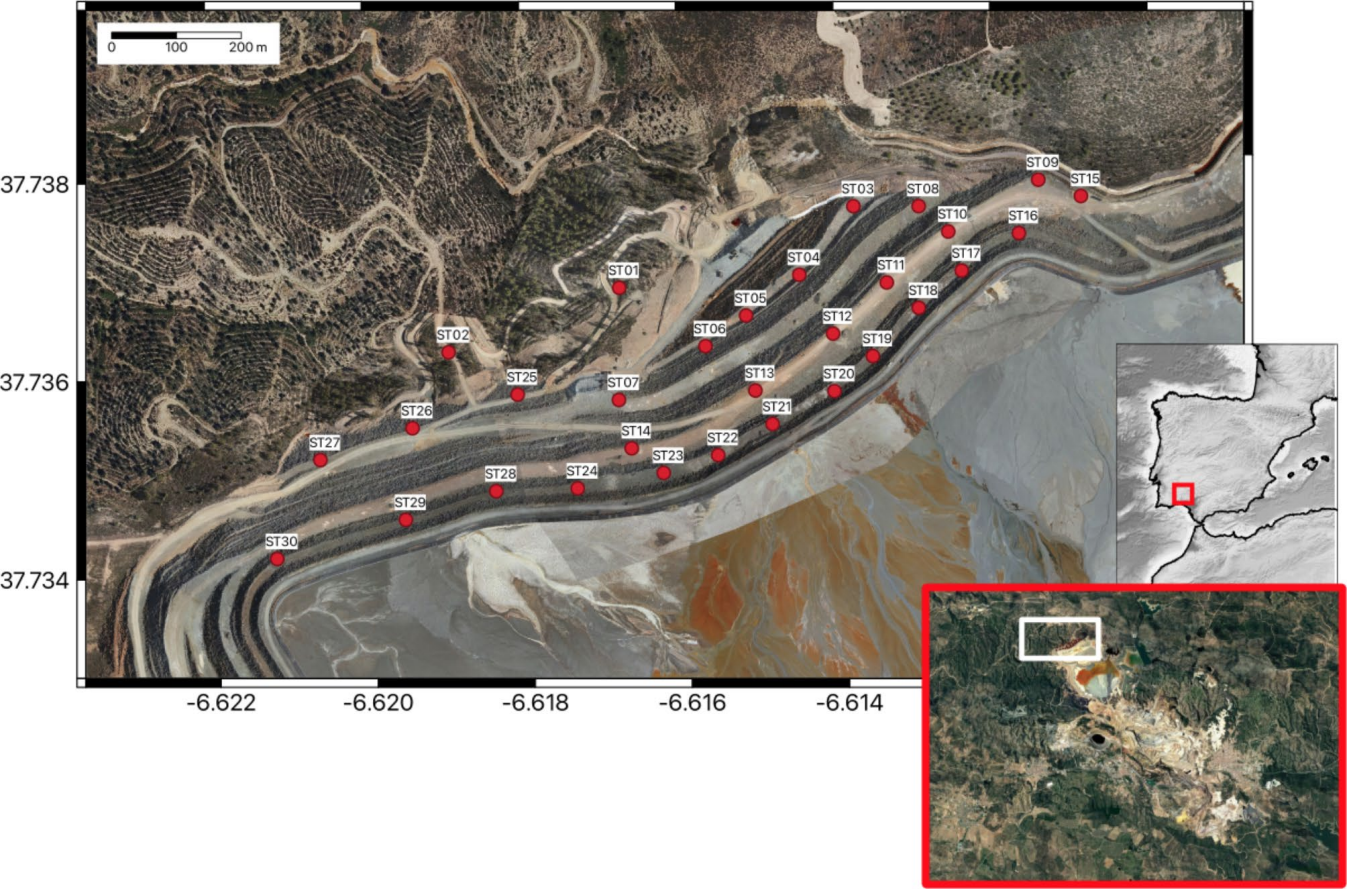
Location of the 30 seismic stations installed at the “Vaguada Norte” dam delimiting the Aguzadera deposit. The insets show the location of the Riotinto mine in southwest Iberia (red box) and the location of “Vaguada Norte” area in the Riotinto complex (white box). Maps are generated using the QGIS software (Version 3.16.14, https://www.qgis.org/ ). The background orthoimage is based on OrtoPNOA 2024 CC-BY 4.0 scne.es, with modifications by Atalaya Mining.

A network of up to 30 seismic stations has been installed in the northern section of the dam, covering an area of approximately 0.18 km^2^ for a period of about six months (December 2022 – May 2023), although six of the instruments remained in operation for five additional months. Figure 1 shows the location of the seismic stations distributed at different levels of the dam, with a couple of additional sites located outside for reference (ST01, ST02). Specifically, the monitored area corresponds to the “Aguzadera” tailings deposit, with the stations located in its NW sector within the area known as the ‘Vaguada Norte’ (Northern trough).

Most of the seismic instruments were provided by the Labsis facility of Geosciences Barcelona (GEO3BCN–CSIC) and were equipped with triaxial Geospace sensors with flat frequency response extending to 2 Hz and GPRS modems, sending data in near real time to our datacenter. The network was completed with 6 Reftek dataloggers with triaxial Mark L22 seismometers, also with a corner frequency of 2 Hz, provided by the Earthscope Primary Instrument Center (EPIC; formerly The IRIS PASSCAL Instrument center).

**Fig. 2:**
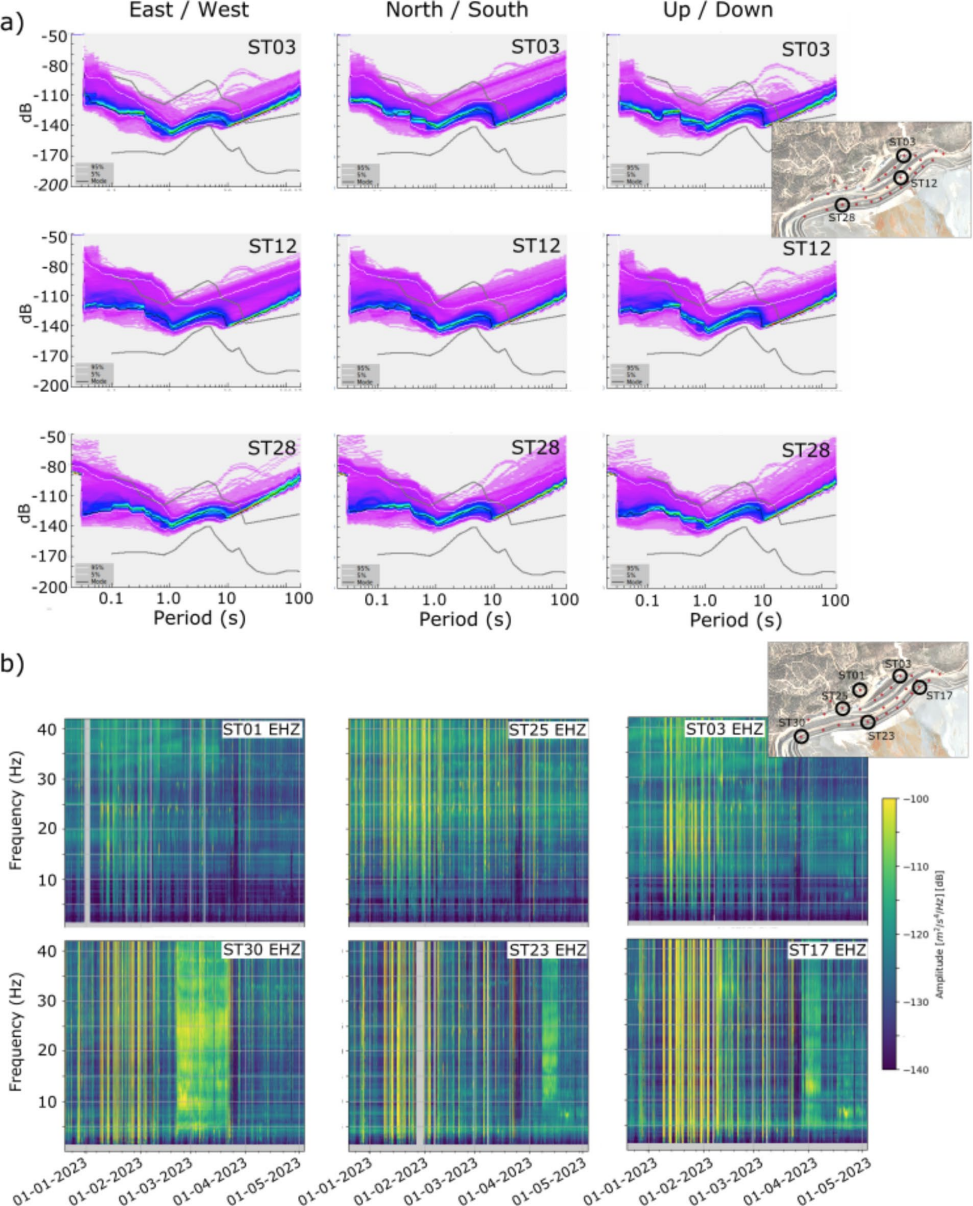
Probability Density Functions (a) and spectrograms (b) of the entire investigated period (December 2022 – May 2023) for representative stations covering different zones of the network. The location of the stations is shown in the inset map (black circles). In (a), each row shows one station, and the columns show the three seismic components (east/west, north/south and up/down).

In order to study the seismic wave field in the Riotinto mine, we first correct the instrument response of the seismic recordings. The background noise power spectral density (PSD) of the vertical components is calculated in one-hour long intervals and statistically analyzed to compute probability density functions (PDFs) using the PQLX software package^12^. This allows to identify stations with anomalous noise levels due to tilting, battery problems etc. and to have a first insight into background seismic noise variations associated with weather, season or time of day. The PDFs show the dominant power amplitude for each period and allow to compare the noise level at each station with the reference NHNM and NLNM models^13^. Figure 2a shows three examples of the obtained PDFs. Since the sensors used are short period, the PDFs only extend to periods of about 10 s. However, the so-called microseismic peak, the frequency band between 1 and 10 s generated by oceanic waves^14^, is clearly identified. At high frequencies, most of the one-hour long intervals have power amplitudes ranging between the Peterson model, with a limited number of cases showing higher values.

In a second stage, we use the SeismoRMS Python package^15^ to extract detailed information of the time varying amplitude at different frequency bands. The data is divided into 30-minute windows with 50% of overlap and the PSD of each window is computed using the Welch method^16^. The results are then used to calculate the spectrograms of the signals, which represent the power of the seismic acceleration, expressed in decibels (dB) and referred to 1 m^2^/s^4^/Hz. As shown in Figure 2b the spectrograms clearly show a large variability with time and frequency. For a single station, the characteristics of the spectra change significantly with time, including periods dominated by large day/night variations or time intervals dominated by a strong signal encompassing all the spectra.

## Characterization of transient seismic waveforms of different origin

Short duration events, such as earthquakes, blastings or vehicles moving near the seismic station can produce high amplitude signals. Although the methods to analyze time variations of the ambient noise account for the eventual presence of such events, it is helpful to review their characteristics before analyzing the properties of the background seismic variations.

**Fig. 3:**
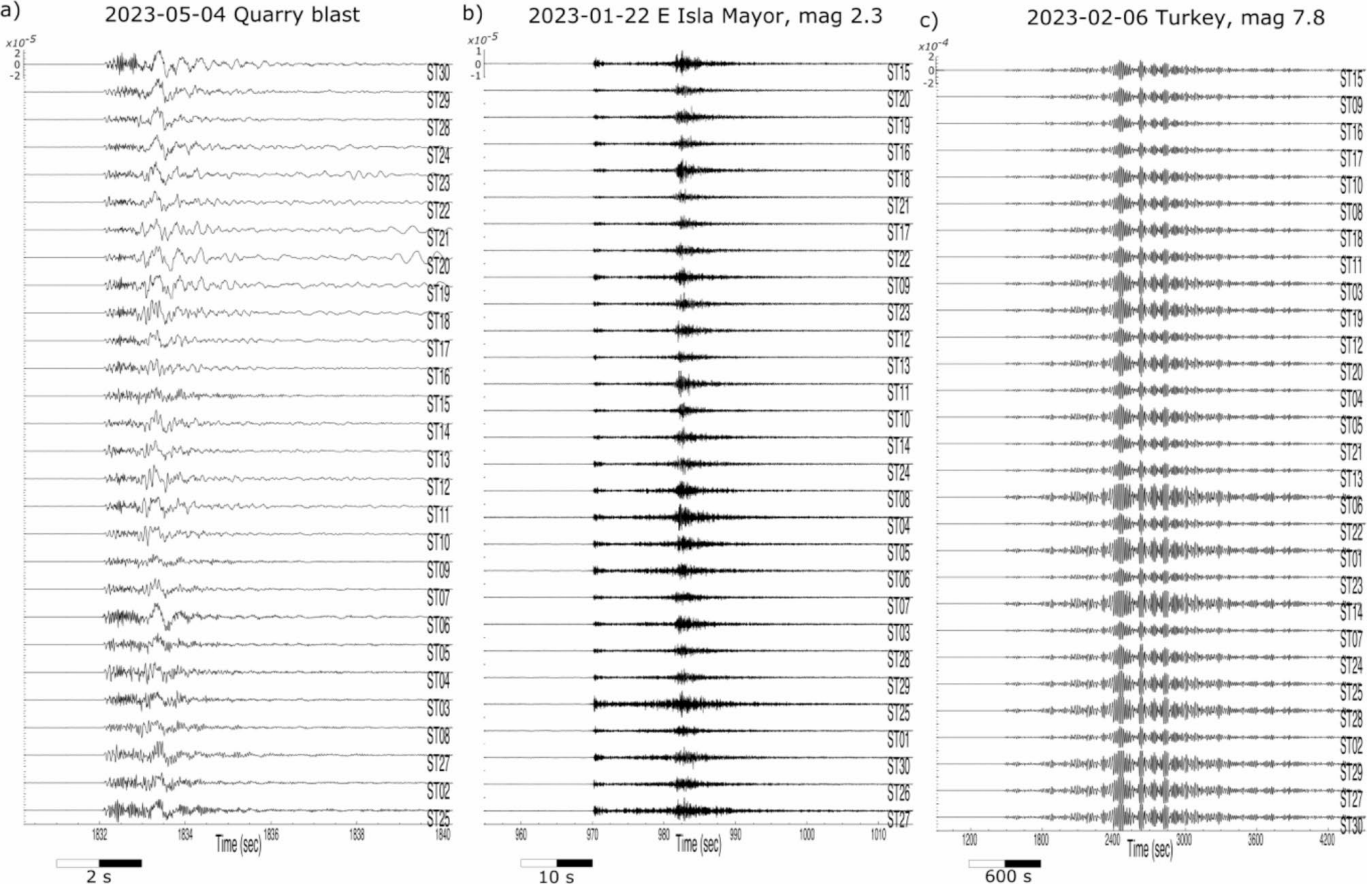
Waveforms corresponding to the record of a local blast (**a**), a local earthquake of magnitude 2.3 with epicenter 83 km away from the network (**b**), and the magnitude 7.8 earthquake with epicenter in SE Turkey, at a distance of 3820 km (**c**). In the latter case, waveforms have been band-pass filtered between 0.01 and 0.1 Hz. Amplitudes expressed in m/s are shown for the first trace of each event.

The signals generated by the blasting periodically detonated at the Riotinto mine as an essential part of the mining activity are one of the dominant transient signals recorded in the network. The active zone of exploitation during the seismic station deployment was located at a distance of about 4.5 km in a SE azimuth. The blasts are typically detonated 5 days a week around midday, and may include two independent shots. To optimize the fragmentation, each shot is composed by up to 200–300 individual blastholes covering areas of around 2500–4000 m^2^ and detonated with microsecond delays. The seismic records of these events show complex signals of short duration (2–4 s) including body and surface phases and have peak velocities close to +/- 3 10^−5^ m/s. An example of this kind of signals is shown in Figure 3a.

Earthquakes located in the Betics Cordillera, the Gulf of Cadiz, the Alboran Sea and Northern Morocco are also regularly recorded by the network. The IGN catalogue (https://www.ign.es/web/sis-catalogo-terremotos) includes around 1330 events within a distance range of 300 km from Riotinto during the investigated time period, 136 of them of magnitude greater than 2.5 and two of them with magnitudes greater or equal than 4.0. The waves generated by these earthquakes are recorded during time intervals which depend on their epicentral distances, but that range between 20 and 30 s to 1–2 min. As an example, Figure 3b shows a magnitude 2.3 event located south of Sevilla at a distance of about 80 km from the mine. Its peak velocity is in the order of 5 10^−6^ m/s, a value clearly below the quarry blast discussed above. The largest regional event recorded, with magnitude 4.2 and located south of Lisbon, 275 km away from our network, has been recorded with peak velocities around 1.5 10^−5^ m/s.

Regarding distant earthquakes, the USGS catalogue (https://earthquake.usgs.gov/earthquakes/search/) includes 129 events with magnitude greater than 5.5 during the same time period, with the largest one being the magnitude 7.8 earthquake that occurred on February 6, 2023 in central Turkey. As for local events, its detectability depends on the background noise, but the seismic waves generated by large amplitude distant earthquakes can be recorded by our short period sensors for time intervals up to one hour. Figure 3c shows the signals of the magnitude 7.8 earthquake in Turkey, 3850 km away from Riotinto. The signals of this event are those showing larger velocities, with peak velocities reaching 2 10^−4^ m/s, exceeding by more than one order of magnitude the signals generated by the quarry blasts. However, if the data is represented in terms of acceleration, as it is usually the case in engineering studies, the amplitude of the teleseismic event decreases significantly due to the relative lack of high frequency energy in this type of signals. Supplemental image S1 presents a visual comparison of the amplitudes of the quarry blast, the Turkey earthquake and some local events, represented in acceleration, velocity and displacement for station ST11.

**Fig. 4:**
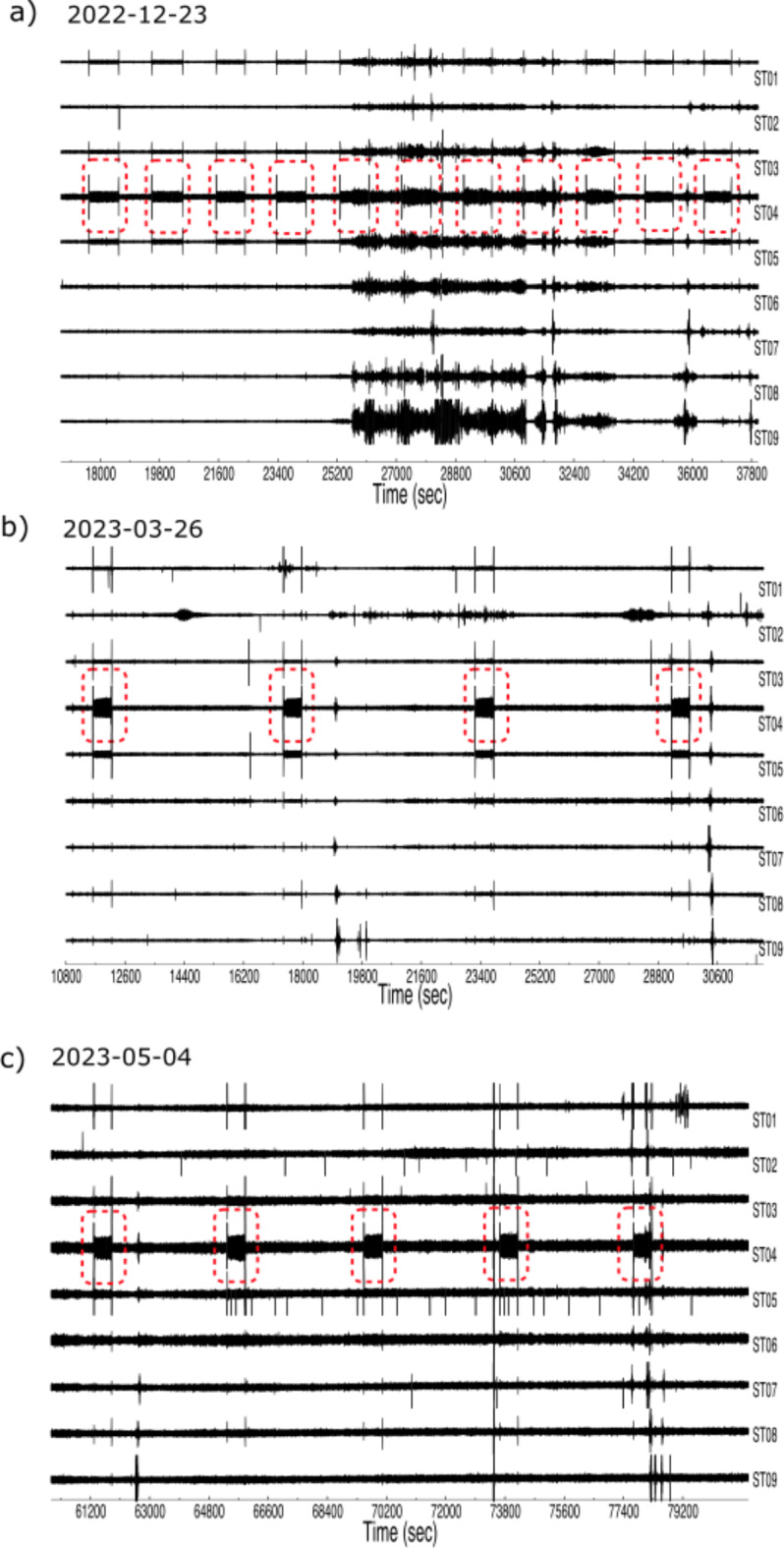
Examples of the signals generated by a water pump, showing the varying pace of the sequence; (**a**) 23/12/2022 (**b**) 26/03/2023 (c) 04/05/2023.

Thoughout our entire seismic dataset, we observe a recurring signal characterized by having short-duration, high-amplitude pulses at the beginning and the end of the signal (Figure 4). The feature lasts a variable time, in the order of a few minutes, and the pulses marking its beginning and end last 1 and 3 s respectively. This feature can be identified in most of the stations during quiet periods such as week-ends, although the feature is clearer at the stations located in the central and NE part of the dam. In between these signals, the seismic record is dominated by a 25 Hz frequency signal, with maximum amplitudes at ST04 and also identified at ST05, ST03, ST01 and ST12. The duration and cycling period of these signals are variable; while on December 23, 2022 the feature lasts 15 min and repeat every 32 min (Figure 4a), on March 26, 2023 the feature lasts 9 min and repeats every 97 min (Figure 4b) and on May 4, 2023 it lasts 9 min and repeats every 70 min (Figure 4c).

Based on the characteristics of the signal and the location of the stations with clearer detections, this feature is thought to be due to the water pumping system operating at the base of the dam to recover leaked fluids, located about 150 m from the seismic station. The water pump is triggered by a sensor, activating or deactivating it as the water level in the reservoir reaches a predefined threshold, which is determined by the spillway elevation. The large transient pulses at the beginning and end of each pumping sequence correspond to the engine switching on and off respectively. The lower amplitude signal with a characteristic frequency of 25 Hz detected in between the pulses can be attributed to the engine vibration and/or to moving water. Stations ST28, ST29 and ST30 show a similar feature, but following a different activation sequence. These signals are interpreted to correspond to a second pump located near the main Aguzadera dam wall, 500 m southward of site ST30, and also triggered by a water level sensor.

**Fig. 5:**
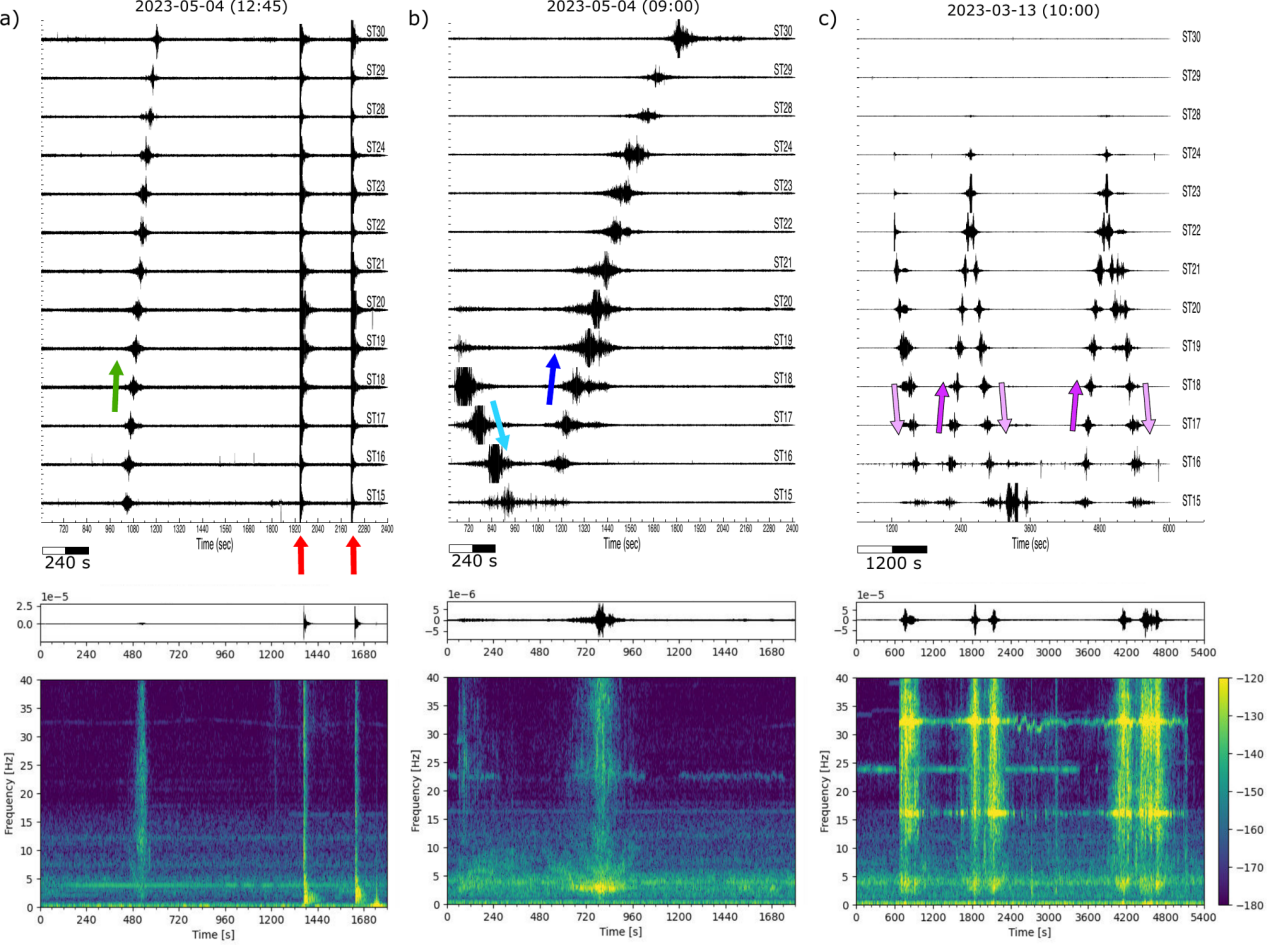
Examples of the seismic signals generated by moving vehicles (**a**) 2023-05-04 (12:45 UTC) Vehicle moving to the SW at 20–25 km/h (green arrow), followed by the recordings of two quarry blasts (red arrows, one of them reproduced in Figure 3a). (**b**) 2023-05-04 (09:00 UTC) Slow vehicles in opposite direction, moving at speeds below 10 km/h. (cyan arrow to the NE, blue arrow to the SW). (**c**) 2023-03-13 (10:00 UTC) slow moving vehicles in both directions (light and dark purple arrows). Lower panels show the waveform and the spectrogram for station ST20. Seismic traces have been filtered between 1 and 40 Hz.

Another major source of transient, short-duration signals is the transit of vehicles along the dam. These signals can be easily discriminated from other sources by the apparent velocity of the signals, that correspond to the speed of the vehicles moving along the dam. Figure 5a compares the amplitude of the signals generated by a moving vehicle near 12:45 UTC on May 4, 2023, whose maximum amplitudes reach 2 10^−6^ m/s, with the blastings detonated at about 4.5 km of distance (the first of this blastings is shown in detail in Figure 3a). The source moves towards the SW with an apparent speed of 20–25 km/h, estimated from the distance between stations and the arrival time at each station. This speed is below the maximum driving speed of 30 km/h within the mine. The corresponding spectrogram (Figure 5a, lower panel) shows energy up to 40 Hz, without indication of harmonics. These properties suggest that the signal is probably generated by a car or a light truck. Figure 5b shows two slow-moving vehicles travelling with opposite directions at 09:00 UTC on the same day. The first vehicle starts from the vicinity of ST18, moves to the NE at an apparent speed of 3–4 km/h and has maximum amplitudes around +/- 5 10^−6^ m/s, twice as large than the vehicle of previous example. The second vehicle moves from NE to SW with a speed slightly higher, but clearly beneath 10 km/h. The spectrogram shows energy crossing all the spectra, but the passage of the vehicle strongly increases the energy at about 3 Hz. Finally, Figure 5c shows multiple movements along the dam on March 13, 2023 between sites ST28 and ST15. In this case, the moving sources have low speeds around 3–6 km/h and high amplitude, with maximum velocities around 1 10^−4^ m/s, almost two orders of magnitudes above the examples in Figures 5a and b. This suggest that the signals belong to some kind of heavy machinery moving along the dam. The spectrogram of this signal covers the entire spectrum, but with three strong harmonics at frequencies of 4, 16 and 32 Hz from the moment in which the moving vehicle is first detected. The presence of these strong harmonics, the low speed and the high amplitude suggest that this signal is generated by a heavy tracked vehicle^17–19^.

## Time variations of the spectral content

Several studies have shown that the origin of the seismic ambient noise is very different across the seismic spectra. Below 0.01 Hz, the signals are dominated by the seismic hum and the Earth tides. The microseismic peak (0.05–0.8 Hz) is mostly generated by the oceanic wave interaction. Above 1 Hz, the dominant sources of noise are the human activity and natural phenomena as wind, rain or floods^20^. Therefore, to characterize the sources of noise in our region of interest, we need to explore different frequency bands. As we are using high frequency sensors and we expect that most of the seismic noise would be related to human activities, we discard frequencies below 1 Hz, analyzing the time variation of the seismic amplitude for the 1–10, 10–20, 20–30, 30–40 Hz frequency bands (Supplemental image S2). The amplitude in each frequency band is evaluated every 30 min and represented by a blue line. To enhance the interpretation of the long-term amplitude variations, we calculate the daily mean values of the amplitude during working hours (red line) as well as the weekly mean of the later value (orange line). Although some differences can be observed between the results for each frequency band, they all follow an overall similar pattern, dominated by clear day/night and working day/week-end variations.

**Fig. 6:**
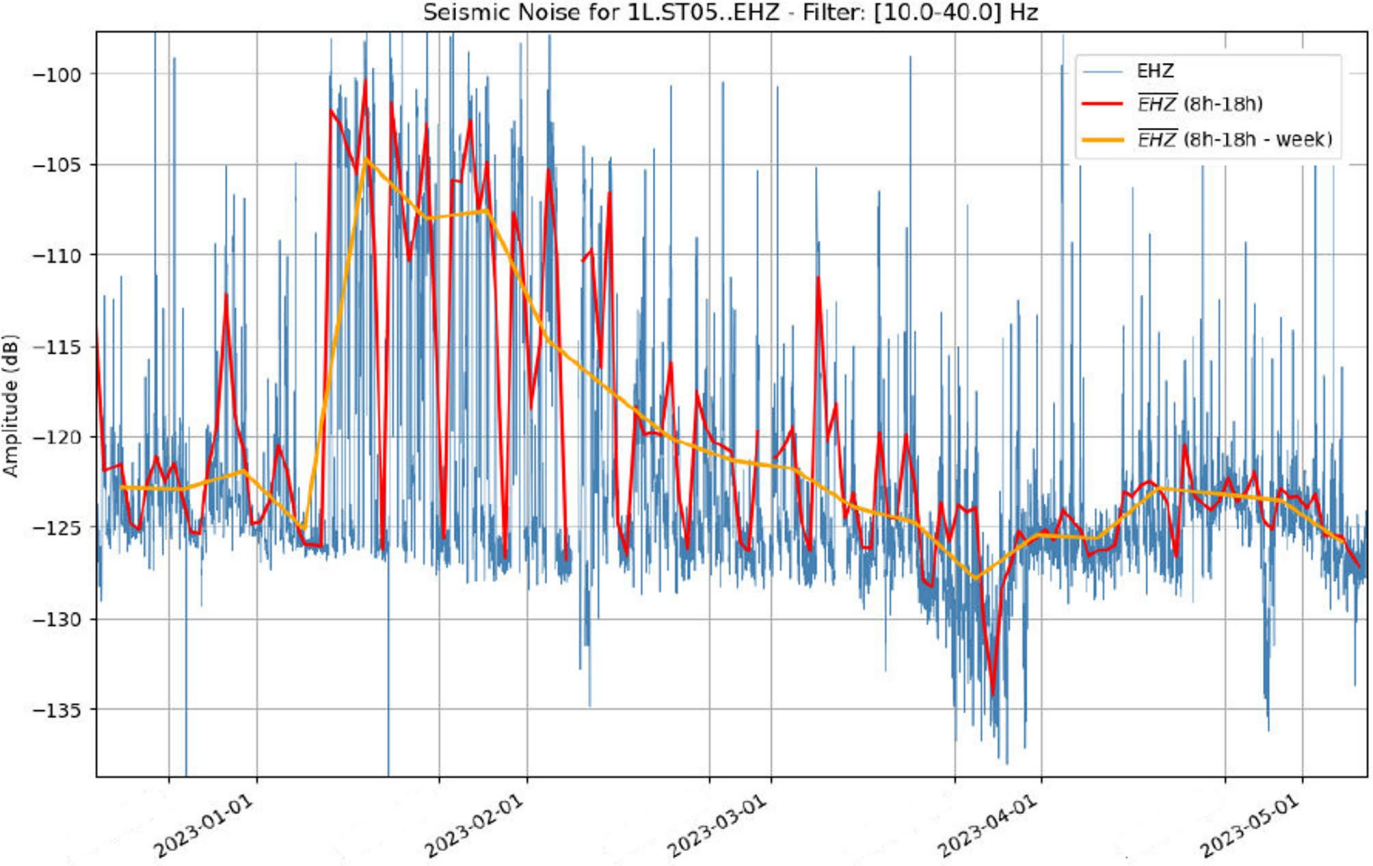
Amplitude versus time changes for the vertical component of station ST03 for the 10–40 Hz frequency band. Blue line: amplitude values every 30 min. Red: mean daily value between 8:00 and 18:00. Orange line: week-averaged value (8:00–18:00).

To further analyze our dataset, we employed the SeismoRMS software^15^, that provides different tools to visualize the time variations of the frequency contents of the signal. The highest amplitudes are recorded from mid-January to mid-February, with a progressive lowering in the following weeks. All the frequency bands show a minimum by late March which is more pronounced at higher frequencies. To work with a representation of the dataset including most of the noise sources identified, we will discuss the frequency variations of the signals in a band extending between 10 and 40 Hz (Fig. 6).

**Fig. 7:**
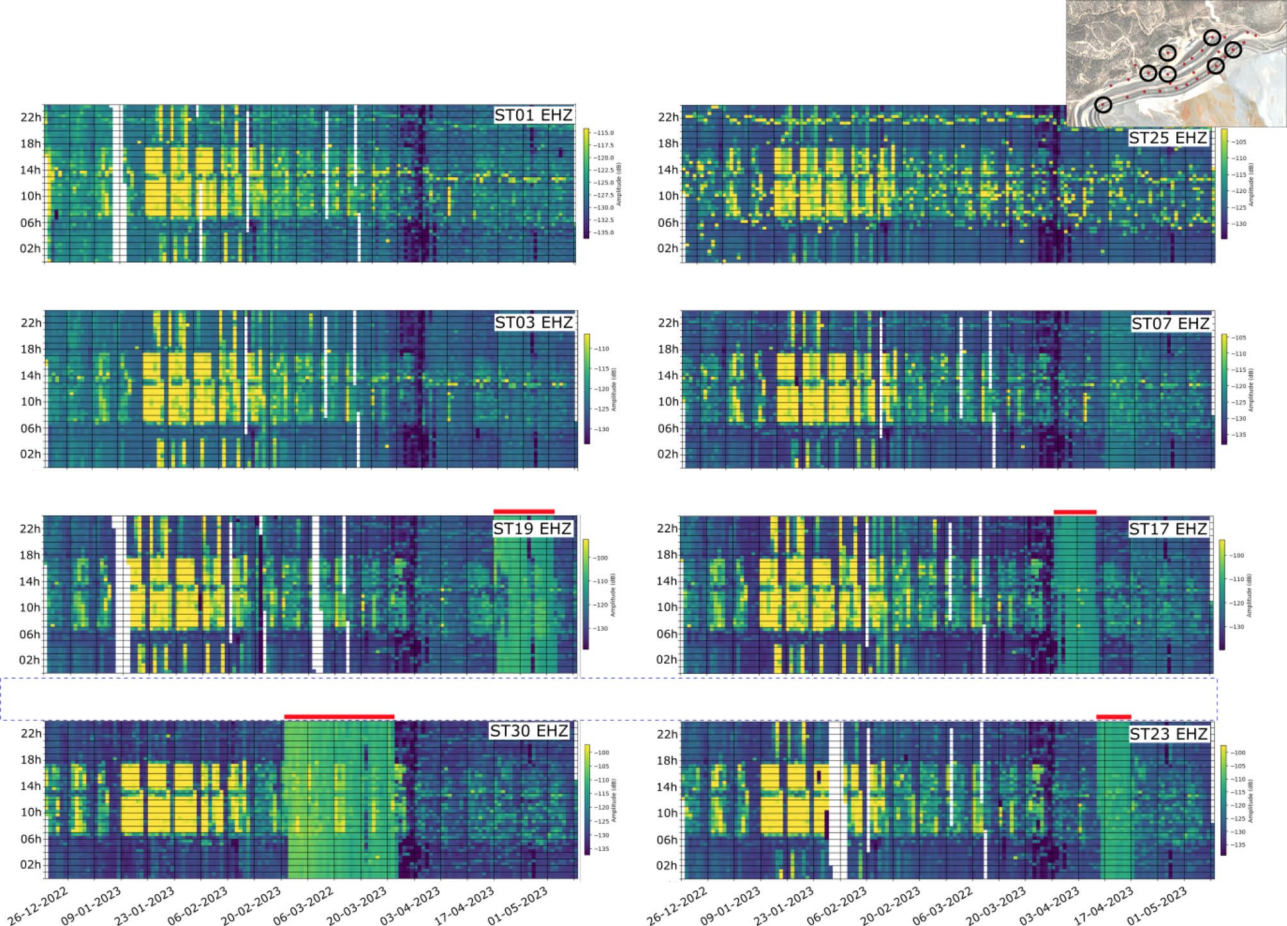
Seismic amplitude in the 10–40 Hz band as a function of recording date and time of the day. Color scale represent the seismic amplitudes in dB. Red lines highlight the periods of sustained noise levels.

Figure 7 shows the amplitude variations as a function of recording date and time of the day for 8 stations distributed along the dam. Note that the recording time is represented in UTC and therefore the change between winter and summer time is observed on March 26 by a shift in the time of the high-noise interval. The dominant feature is the daylight/nighttime variation, with high noise starting around 6:30 and vanishing around 17:30 UTC. This is clearly associated with working hours in the mine, as also evidenced by the lower noise level during the lunch break, between 13:30 and 14:30 UTC. For a limited number of nights during January, high noise extends to the night hours and does not end until 4:30 UTC. While increased noise levels during working hours can be identified during most of the recording periods, it is stronger during January 2023. The week-ends are characterized by a lower level of noise across the whole day. The quietest period, especially during nighttime hours, is recorded in late March, when mining activity appears to have been minimal. This quiet period coincides with a plant shut down for maintenance scheduled from March 22 to 28. Starting by late February, frequency bands of high noise having the same amplitude all the day long can be identified as greenish bands for the stations located near the dam rim (e.g., ST19, ST17, ST30 and ST23) masking the day/night variations. The origin of these sustained sources of noise over time is discussed in the next section.

**Fig. 8:**
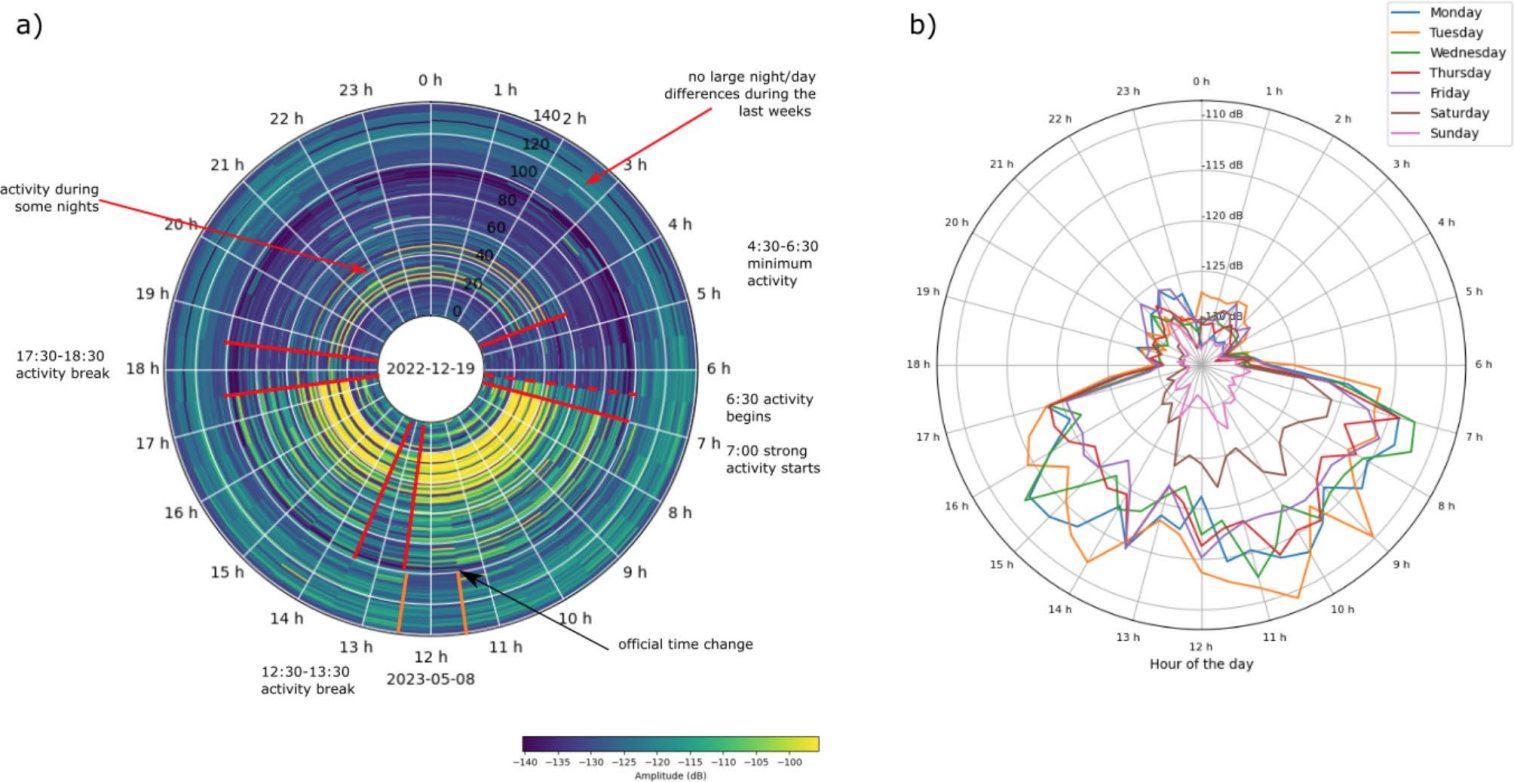
Diurnal and weekly variations of the seismic noise. (a) Seismic amplitude variations as a function of date (radius) and hour of the day (azimuth) for station ST21. Color scale represents the seismic amplitude in dB. Each circle is for one day, with the starting date (2022-12-19) in the center and the last data (2023-05-08) in the outer ring (for reference, white circles mark 10 days intervals). (b) Weekly amplitude variations for the same station and time period from a). Each colored line shows the mean values per week day. Hours are reported in UTC.

Figure 8a, which represents the amplitude variations as a function of date and hour of the day for station ST21, allows us to better visualize the start of activity in the mine, the break-off intervals, the change in the official time or the highest noise at night for the last weeks. As observed, the activity begins at 06:30 UTC and significantly increases half an hour later. Lunch breaks are clearly observed between 12:30 and 13:30 UTC and facilitate the identification of the official time change. As commented before, activity during some nights in January can be identified by the yellowish colors shown in Figure 8a. For these days, a break-off interval can be identified between 17:30 and 18:30, reflecting the change of shift of the workers.

Figure 8b shows the noise amplitude at ST21 as a function of the day of the week (colored lines) and the hour of the day (azimuth). From Monday to Friday, the noise variation is similar, with maximum values close to -115 dB between 07:00 and 17:00 UTC. On Saturdays the amplitudes range between − 120 and − 125 dB during the morning, but are greatly diminished after 13:00 h UTC, while on Sundays the noise remains below − 130 dB throughout the day. All these observations confirm that the seismic noise is dominated by human-made activity and is affected by the activity changes in the mine environment.

**5 Time sustained noise sources**.

**Fig. 9:**
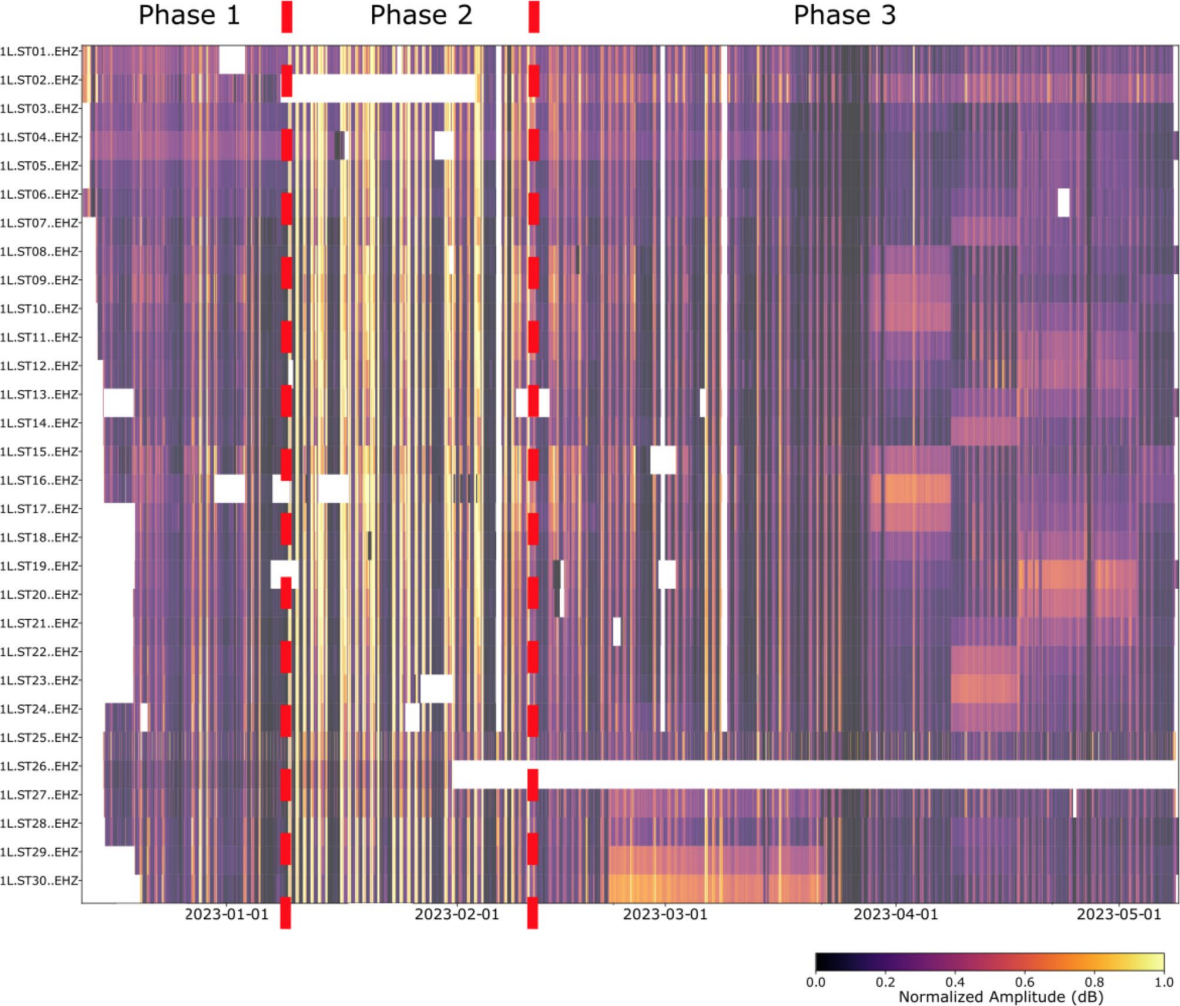
Normalized displacement for all the available seismic stations, with lighter colors showing the intervals with highest noise. The red dashed bars and the corresponding labels identified the three phases discussed in the text.

A global view of the noise variation in the Riotinto mine is represented in Fig. 9, which displays the normalized displacement amplitude throughout the entire recording period for all the available stations, with lighter colors indicating larger values. We identify three distinct phases for the background noise variations in the recordings. Phase 1 spans from the start of our records to January 9, 2023 and it is characterized by moderate amplitudes, day/night variations, lower noise level during weekends and consistent results across all the stations. This phase is interpreted as representing the typical seismic ambient noise in the area. Phase 2, which spans from January 9 to February 10, 2023 is characterized by a large increase in noise during daylight hours including a large number of moving vehicles, while on weekends the noise level remains similar to that of Phase 1. During this period, civil works aimed at increasing the height of the dam were carried out in the area, as reported in the dam operation logs. This confirms that a large part of the ambient noise is related to local activity near the recording stations. Phase 3, extending to the end of the analyzed period, shows day/night variations similar to Phase 1, but is characterized by the presence of time intervals of high noise observed at a limited number of neighboring stations, a feature related to the deposition of tailings, as discussed below.

**Fig. 10:**
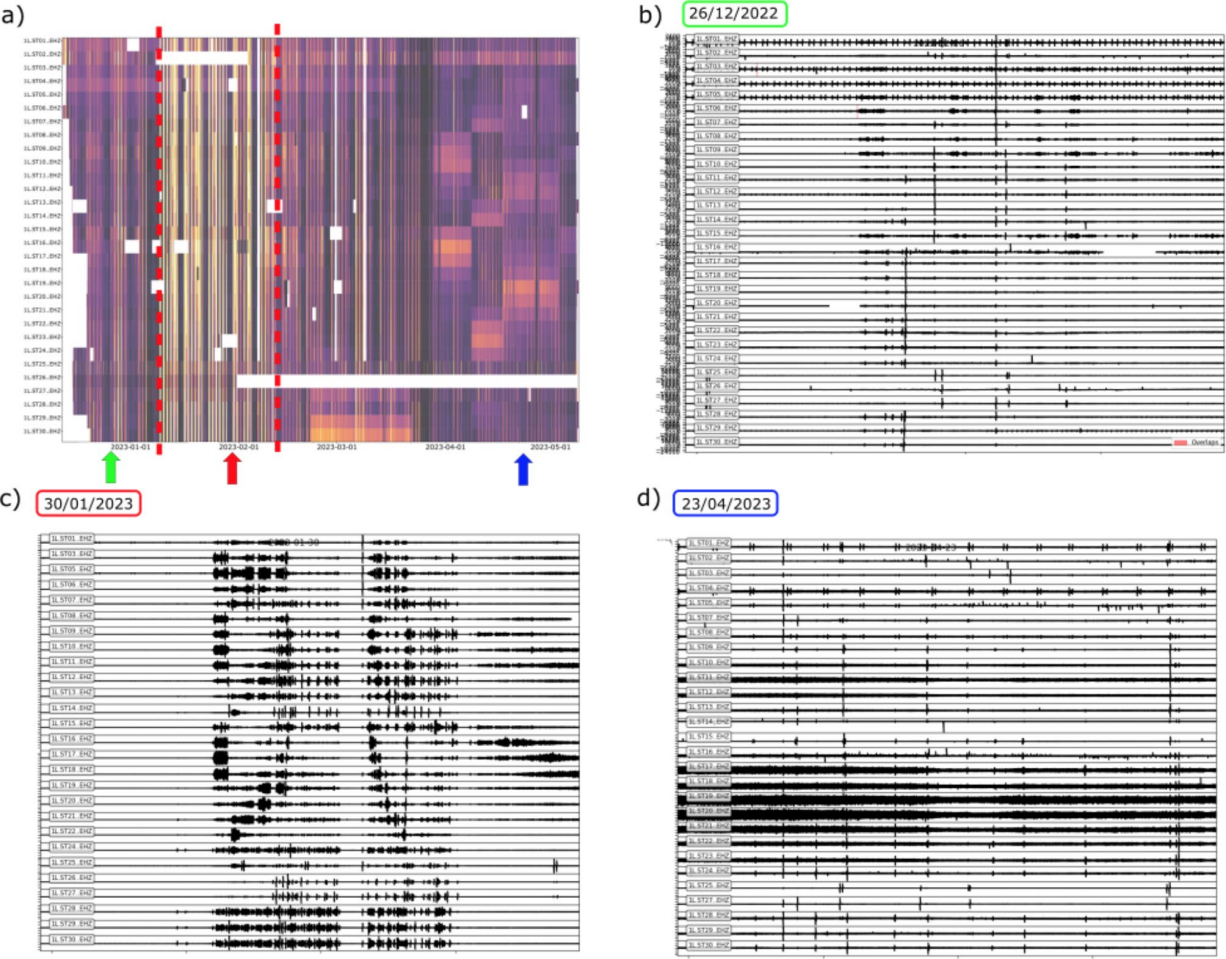
Characteristic waveforms for each of the three defined phases. (**a**) Normalized displacements for all the stations. Colored arrows mark the timings of recordings in panels b-d, lighter colors showing the intervals with highest noise. (**b**) 24 h recording during Phase 1 (December 26, 2023). (**c**) 24 h recording during Phase 2 (January 30, 2023). (**d**) 24 h recording during Phase 3 (April 23, 2023).

Inspection of the waveforms provides a more visual representation of the characteristics of each of the defined phases, as shown in Fig. 10, which displays the waveforms on representative dates for phases 1, 2, and 3, indicated by colored arrows in Figure 10a. The seismic records during Phase 1 show a moderate increase of amplitude during daytime and are dominated by transient signals generated by moving vehicles and blastings, while signals generated by water pumps can be identified during low-noise intervals (Figure 10b). Phase 2 waveforms are dominated by the vibrations due to the machinery and vehicles involved in the dam’s regrowth process. This results in a strong contrast between working hours and non-active intervals, including the lunch break, that is clearly defined in the waveform amplitude variation (Figure 10c). Finally, Phase 3 is characterized by a high level of seismic noise, active over the entire 24 h period and detected only at groups of stations, (e.g., ST17 - ST21 on April 23, 2023; Fig. 10d).

**Fig. 11:**
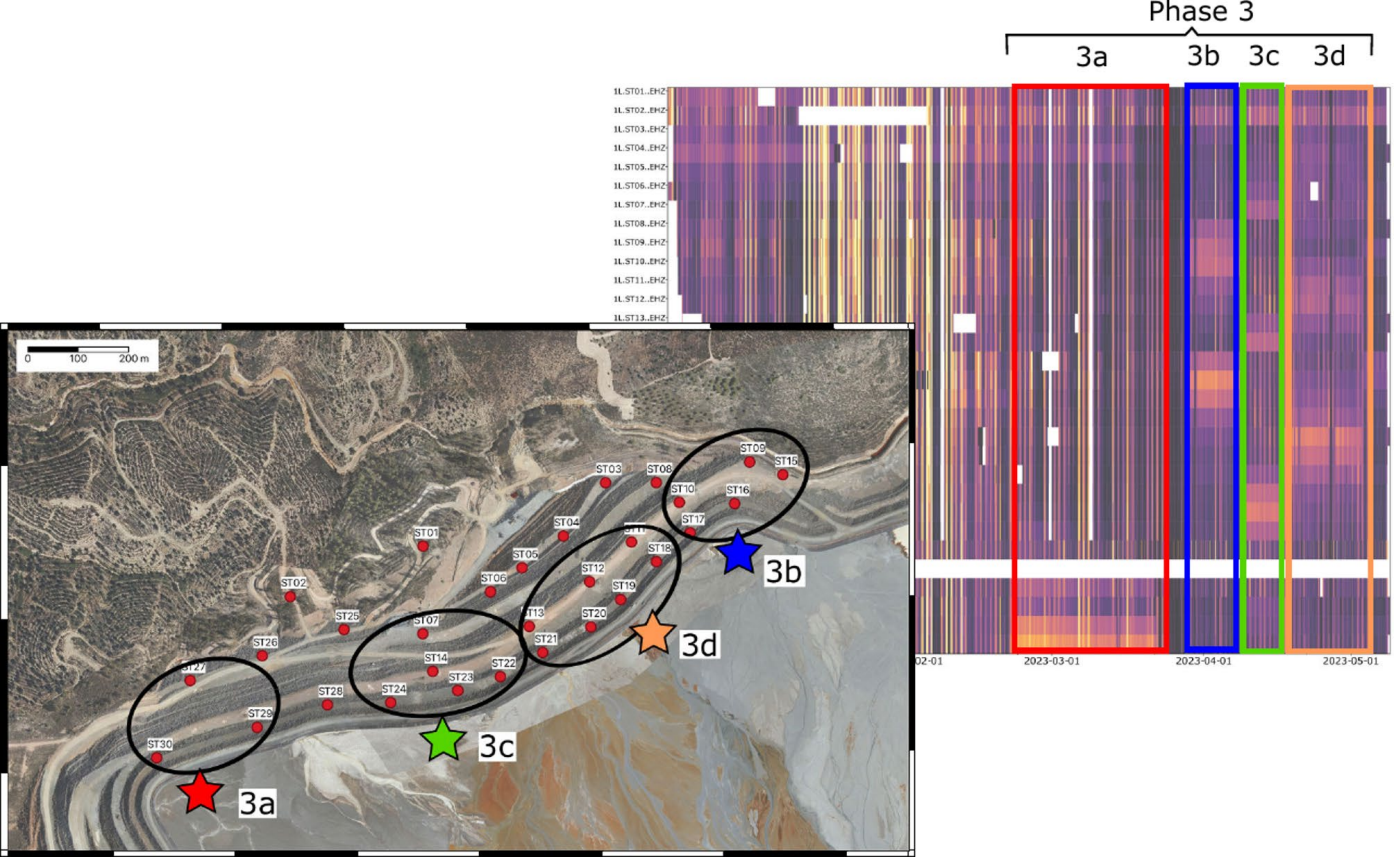
Active tailings deposition sites as indicated by mine activity logs (color stars) and location of the seismic stations with highest ambient noise amplitude (lighter colors) during phases 3a to 3d (black circles). Map is generated using the QGIS software (Version 3.16.14, https://www.qgis.org/ ). The background orthoimage is based on OrtoPNOA 2024 CC-BY 4.0 scne.es, with modifications by Atalaya Mining.

During Phase 3 the subset of stations with the highest amplitude at each time period, allows us to define four distinct subphases, represented by colored boxes in Figure 11. During this time period the mine restarted the deposition of tailings in the Aguzadera pond, using different deposition locations to uniformly distribute the tailings. Phase 3a begins on February 21 and remains active until March 22. This is observed at sites ST30, ST29 and ST27, located at the SW end of the dam. After a few days without tailings deposit activity, Phase 3b begins on March 28 and ends on April 8, 2023. During this phase, stations ST16, ST17, ST10, ST09 and ST15, all of them located at the NE termination of the dam, show the highest amplitude. Phase 3c begins immediately after and shows the largest amplitudes at sites ST23, ST24, ST22, ST14, and ST07. Finally, Phase 3d extends between April 17 and May 1 and is identified at sites ST19, ST20 and, to a lesser extent, at ST11, ST12, ST18 and ST21. The location of the sites with the greatest amplitudes in each subphase is totally consistent with the position of the deposition points that are active in each time interval, as recorded in the mine activity records. These sites are represented with colored stars in Figure 11.

**Fig. 12:**
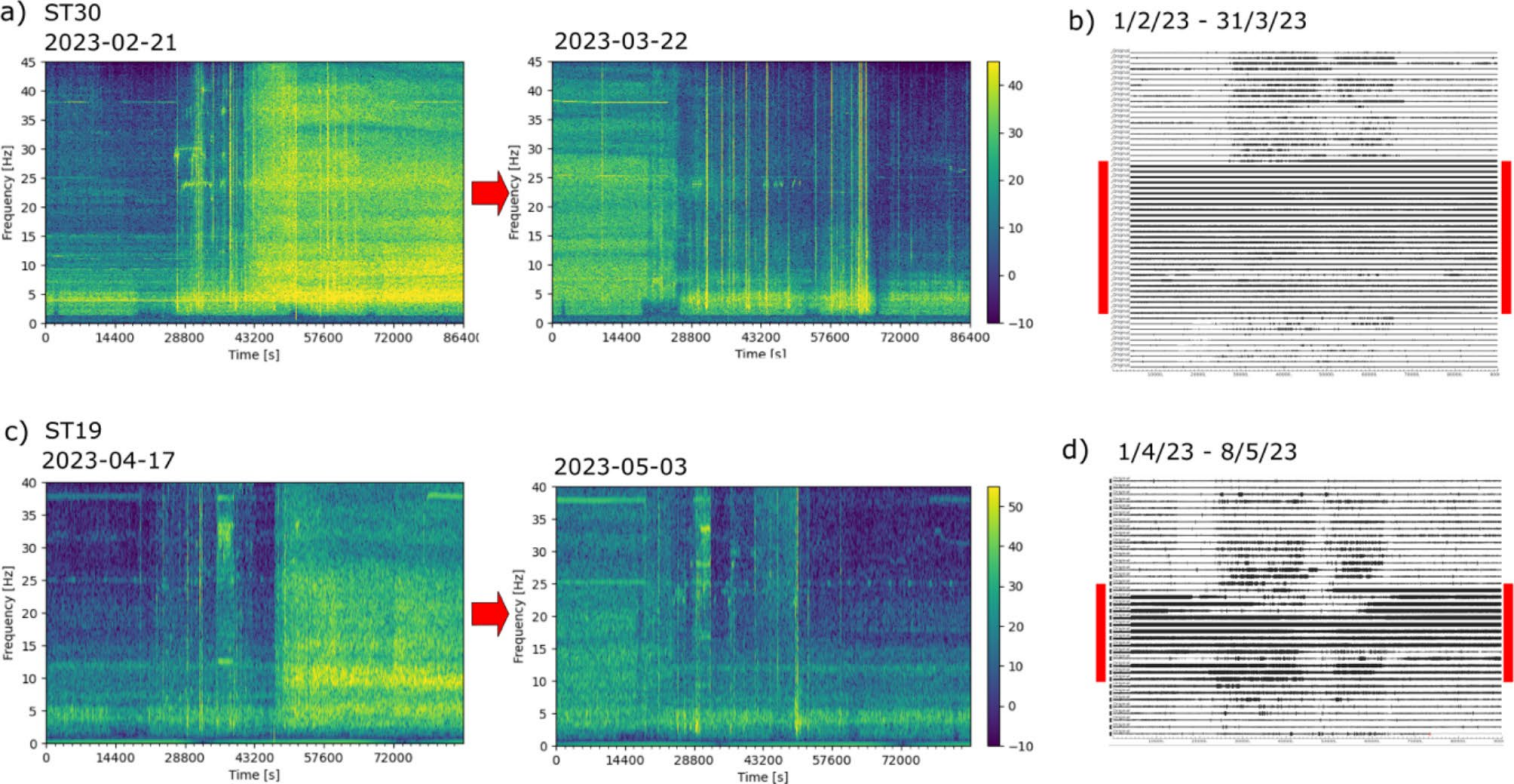
Identification of the signals related to tailings depositions. (**a**) Spectrograms for the first and last day of Phase 3a, as recorded at station ST30. (**b**) Seismic waveform (one day per line) at ST30 between 1 February and 31 March 2023. Red bars show days with active tailings deposition.** c**), **d**) Idem for the Phase 3d recorded at ST19. In this case waveforms from 1 April to 8 May are shown.

The inspection of the spectrograms of the seismic data makes it possible to precisely define the beginning and end of each deposition sequence (Figure 12 and Supplemental images S3a-d). Although the deposition process is typically continuous, there are occasions when it becomes necessary to interrupt the process for technical reasons. The seismic observations allow us to monitor in detail these disruptions, which are not always reported in the mine logs and can vary in duration from 8 to 30 h. As observed in Figure 12, the 5–25 Hz frequency band is the most useful to be used as a proxy of the tailings deposition process.

## Conclusions

The in-depth analysis of the seismic recordings acquired during the 6 months-long deployment at a tailings dam in the Riotinto open-pit mine has allowed us to identify the most common sources of seismic noise. These include transient signals generated by earthquakes, blastings, water pumps, moving vehicles or machinery, but also more continuous features, as the deposition of tailings.

Blastings occurring 4.5 km away from the stations are recorded with modest amplitudes, similar to that of a magnitude 4.0 earthquake located at 160 km. Water pumps produce recurrent seismic signals with variable duration and cycling period, with a 25 Hz dominant frequency. Moving vehicles and heavy machinery generate seismic signals crossing the entire spectrum including, in the case of heavy machinery, strong harmonics. Time sustained sources of noise are dominated by the vibrations generated by the deposition of tailings in the pond. These different sources can be identified with a low margin of error, since their waveforms and spectral content are clearly different.

We conclude that seismic data should be viewed as a useful tool in mining environments, as it provides a practical way to monitor multiple mining activities at a low cost and with easy implementation. As we have seen, seismic detectors can be used to check the amplitude of vibrations generated by mine blastings, to control the activity of water pumps or to monitor the traffic of trucks and heavy machinery along specific sections of the mine. Although this kind of signals have been used in previous studies, our results pave the way towards the development of machine learning-based methods for the automatic detection and classification of the different transient signals in an active open pit environment, that could improve the monitoring of seismic activity due to mining activity. In addition, seismic data also enables the monitoring of long-term processes, such as the deposition of tailings in a dam. A dense array of seismic stations allows precise identification of the location of the deposition points active at each time interval. Furthermore, the noise characterization presented in this study provides a solid foundation for follow-up studies on the potential changes in subsurface physical properties using ambient noise seismic interferometry methods, as it will help to discern between changes caused by structural modifications and those due to source variability. Therefore, we suggest that installing of semi-permanent seismic networks in open-pit mining environments may be of interest to the industry.

## Methods

*Seismic waveforms and record section plots are produced using the ObsPy software*^21,22^*and the Seismic Analysis Code (SAC)*^23^.

*Maps are generated using the QGIS software (Version 3.16.14*, https://www.qgis.org/*).*

*Background orthoimages are based on OrtoPNOA 2024 CC-BY 4.0 scne.es, with modifications by Atalaya Mining.*

*Spectrograms are obtained by dividing the signal in overlapping intervals of a given length and calculating the Power Spectral density in each of them*,* using the ObsPy software*^21,22^. *The spectrograms in* Fig. 2* are calculated using a window length of 1800 s and an overlap of 50%*,* while those in* Fig. 5* are calculated with a window length of 120 s and the same overlap rate. In both cases*,* a color palette*,* expressed in dB and relative to a reference value of 1 (m*^*2*^*/s*^*4*^*)/Hz*,* is used to show the energy distribution.*

## Data availability

The seismic dataset used in this study, acquired by the temporary 1L (10.7914/SN/1L_2022, Schimmel, 2022), will be publicly distributed through the Inst. Cartogràfic i Geològic de Catalunya (ICGC) EIDA Data Center (http://ws.icgc.cat/fdsnws, last access: 11 March 2024) at the end of the embargo period. In the meantime, data are available upon request at the Geo3Bcn-CSIC data center (http://eida.geo3bcn.csic.es:8080/fdsnws/, last access: 11 March 2023).

## Electronic supplementary material

Below is the link to the electronic supplementary material.

Supplementary Material 1
