# Supplementary material for: Sources of seismic noise in an open-pit mining environment
Source: Sci Rep. 2024 Oct 15;14:24106. doi: 10.1038/s41598-024-75733-2 (PMC11480340; doi:10.1038/s41598-024-75733-2)

**Supplementary Material**

***Supplementary Figure S1****: Visual comparison of the amplitudes of a magnitude 2.3 local earthquakes located south of Sevilla (distance: 80 km), a magnitude 4.2 located south of Lisbon (275 km), a magnitude 4.0 earthquake located in the Gulf of Cadiz (163 km), a quarry blast at Riotinto (distance :4.5 km) and the m 7.8 6/2/2023 Turkey earthquake (distance 3850km), represented in acceleration (a), velocity (b) and displacement (c) for station ST11.*

***
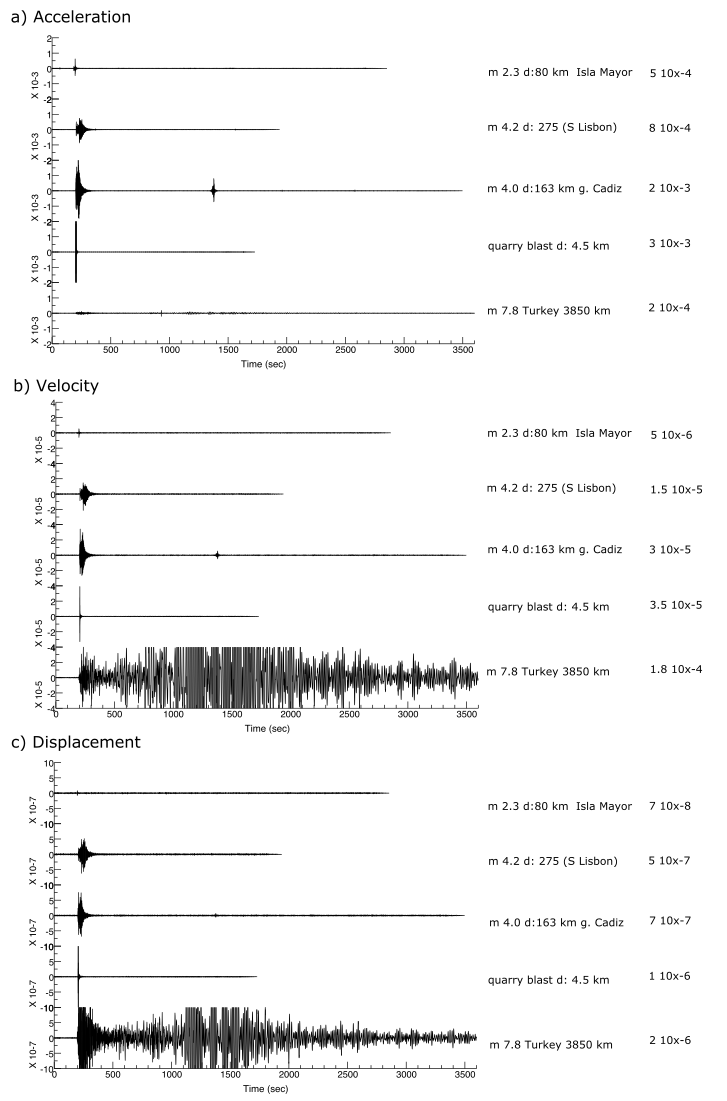
***

***Supplementary Figure S2:*** *Amplitude versus time variation for station ST03 for frequency bands 1-10 (a), 10-20 (b), 20-30 (c) and 30-40 (d) Hz. Blue line: amplitude values every 30 minutes. Red: mean daily value between 8:00 and 18:00. Orange line: weekly value (8:00-18:00)*

*
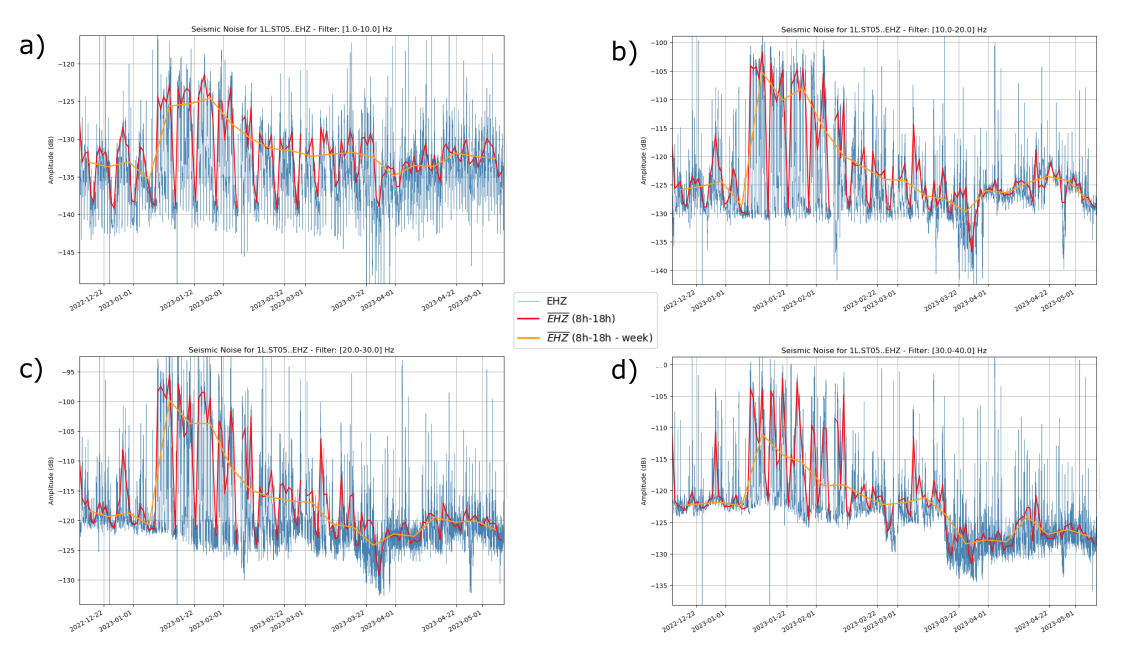
*

***Supplementary Figure S3a:*** *Daily spectra for the seismic station closest to the deposition point for Phase 3a ( ST30)*


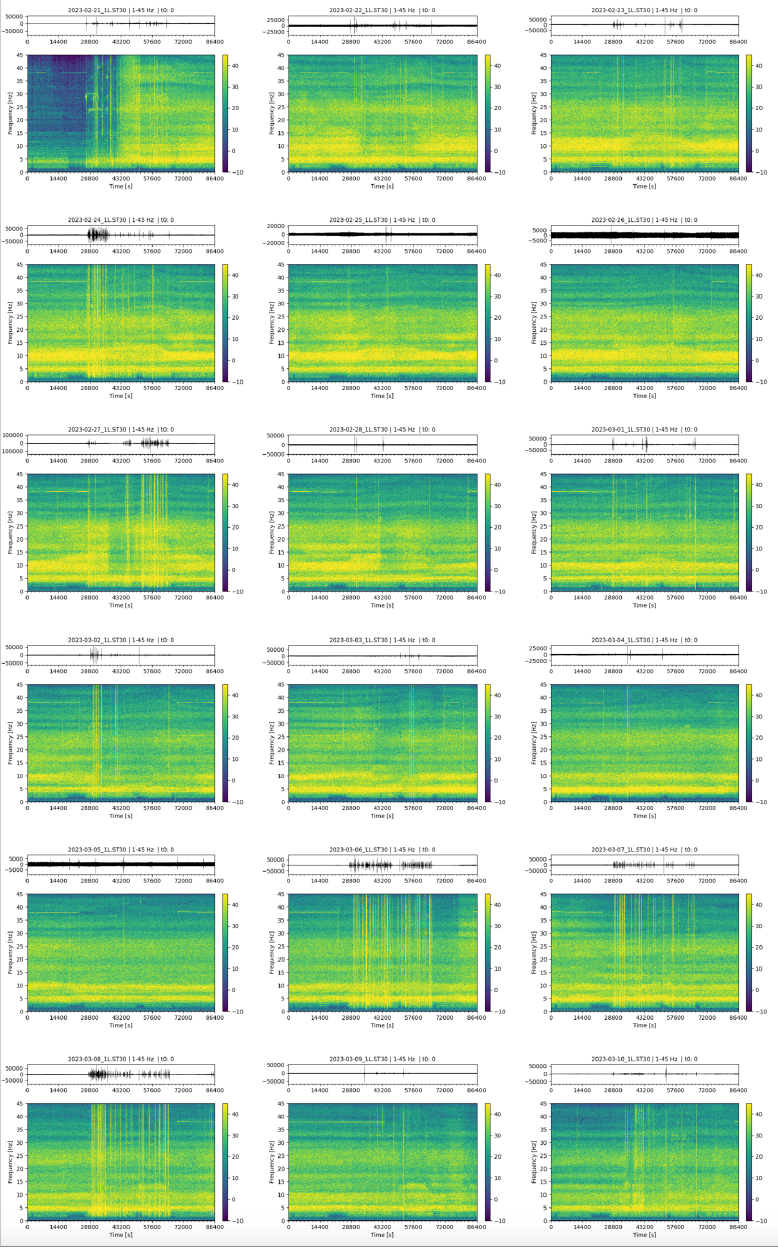


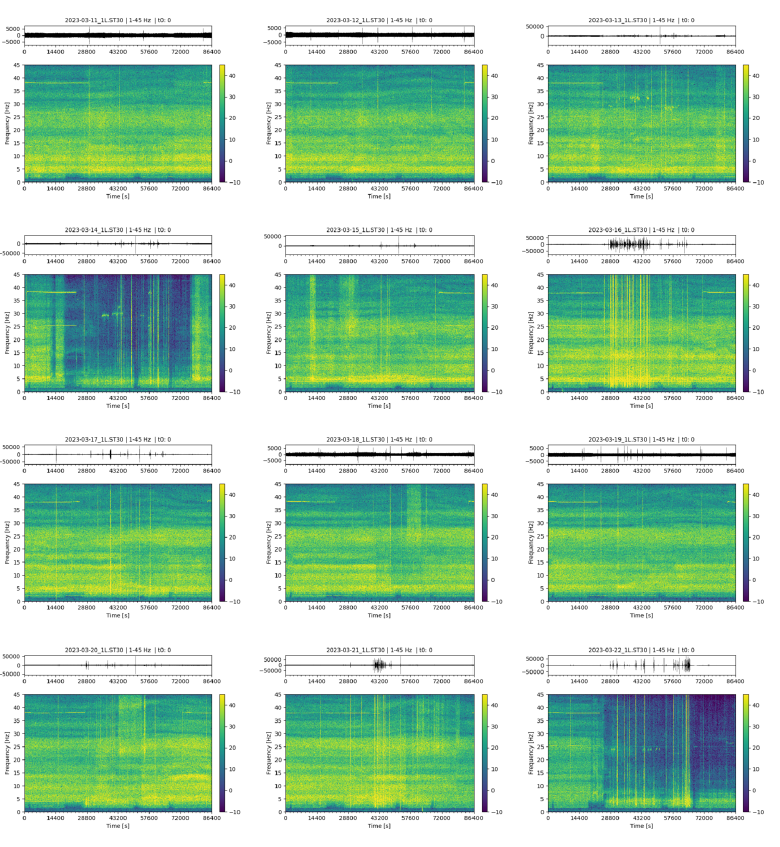


***Supplementary Figure S3b:*** *Daily spectra for the seismic station closest to the deposition point for Phase 3b ( ST16)*

***
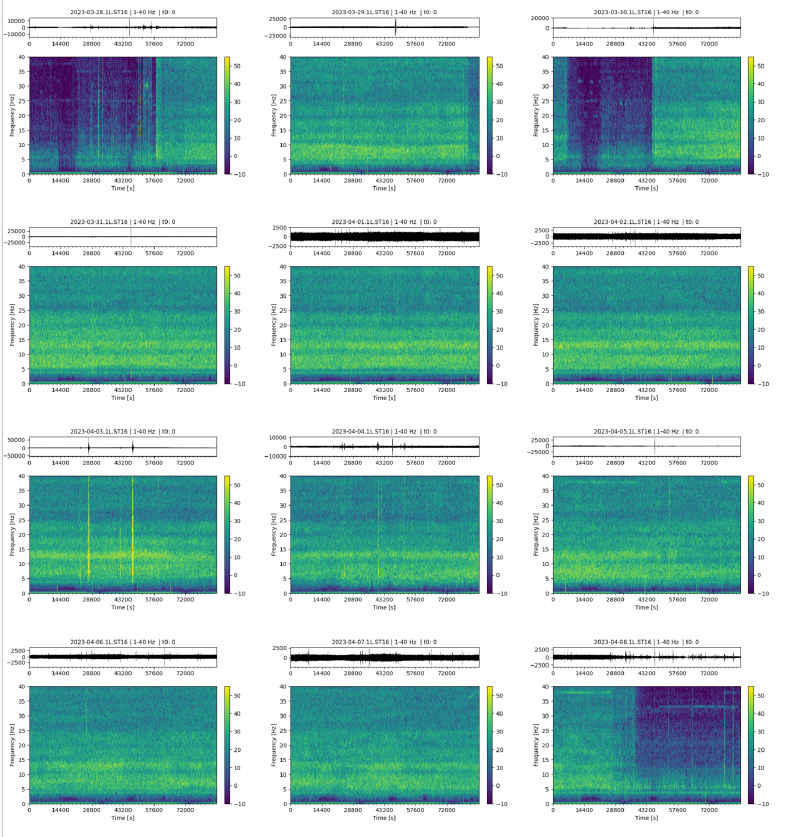
***

***Supplementary Figure S3c:*** *Daily spectra for the seismic station closest to the deposition point for Phase 3c ( ST23)*

*
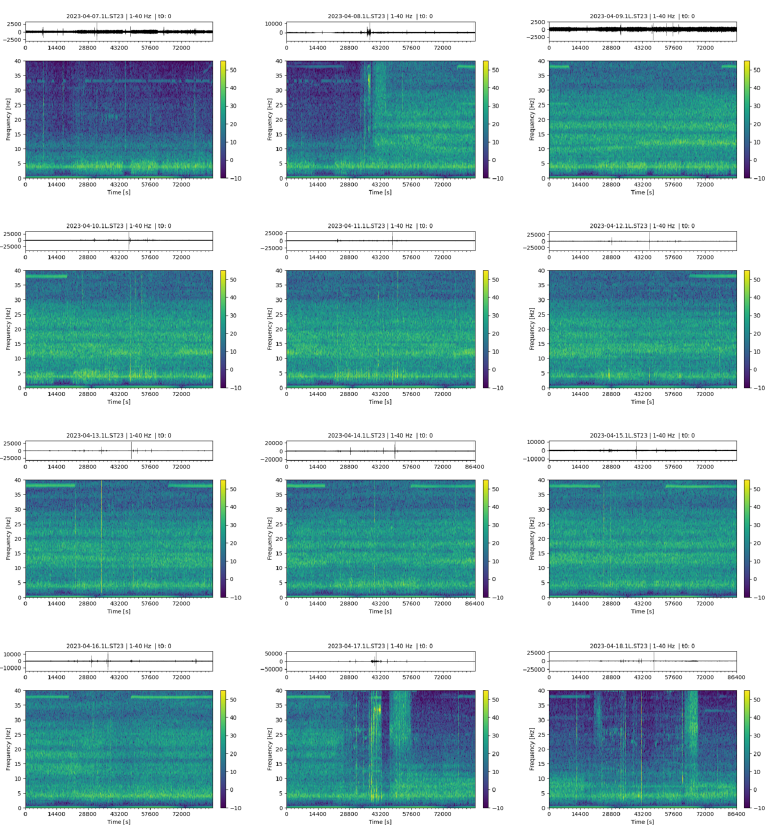
*

***Supplementary Figure S3d:*** *Daily spectra for the seismic station closest to the deposition point for Phase 3d ( ST19)*


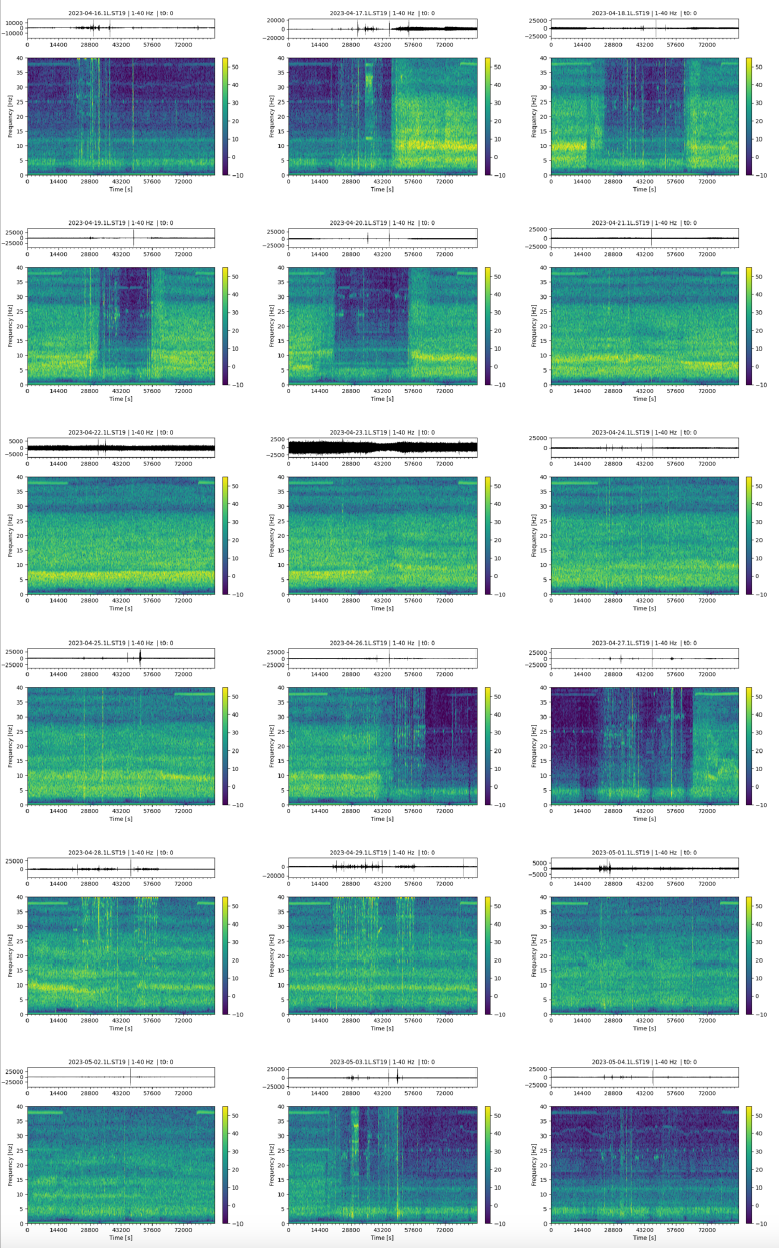

Supplement: Supplementary file 1 — Supplementary Material 1 [file 41598_2024_75733_MOESM1_ESM.docx]
